# Diversity of human papillomavirus typing among women population living in rural and remote areas of Brazilian territory

**DOI:** 10.1016/j.pvr.2019.100186

**Published:** 2019-09-17

**Authors:** Adriana Tarlá Lorenzi, José Humberto Fregnani, Luisa Lina Villa, Laura Sichero, Emily Montosa Nunes, Adhemar Longatto-Filho

**Affiliations:** a Teaching and Research Institute, Molecular Oncology Research Center, Barretos Cancer Hospital, Pio XII Foundation, Brazil; b Teacher at Faculty of Medicine, Alfredo Nasser Faculty (UNIFAN), Goiás, Brazil; c Superintendence of Education of A.C.Camargo Cancer Center, Brazil; d Molecular Biology Laboratory, Center for Translational Research in Oncology, Instituto do Cancer do Estado de Sao Paulo - ICESP, São Paulo, Brazil; e Department of Radiology and Oncology, School of Medicine, Universidade de São Paulo, Brazil; f Medical Laboratory of Medical Investigation (LIM) 14. Department of Pathology, Faculty of Medicine, Universidade de São Paulo, Brazil; g Research Institute of Life and Health Sciences (ICVS), School of Medicine, University of Minho, Braga, Portugal; h ICVS / 3B's - Associated Laboratory to the Government of Portugal, Braga, Guimarães, Portugal

**Keywords:** Cervical cancer, Cervix neoplasms prevention, Genotype, Human *papillomavirus* DNA tests, Self-examination, Screening

## Abstract

**Objectives:**

Genotyping HPV from samples tested positive to careHPV™ assay in rural and remote areas of Brazilian territory.

**Methods:**

A total of 5079 women were enrolled in an opportunistic screening from the Barretos Cancer Hospital, through mobile units or ambulatory unit. All careHPV™ hr-HPV positive samples were tested by a Luminex-based protocol in order to evaluate the HPV infecting types.

**Results:**

Positive hr-HPV results were obtained in 10.6% (536/5068) of women. Among these cases, HPV-56 and HPV-51 were the most common types detected in 32.3% and 31.4%, respectively. HPV-53 (20.5%), HPV-18 (18.5%), HPV-58 (17.6%), HPV-52 (16.0%) and HPV-16.6%) were the other most frequent types detected. These frequencies represent prevalences of 2.35%, 2.12%, 2.02%, 1.84% and 1.80% respectively, within the population studied. Regarding low-risk HPVs, HPV-6 was detected in 12.9% of the samples. The less frequent types (<3%) were: HPV-70, HPV-11 and HPV-26.

**Conclusions:**

The most frequent types detected were: HPV-56, HPV-51, HPV-53, HPV-18, HPV-58, HPV-52 and HPV-16 according to decreasing rates.

## 1 Introduction

Cervical cancer incidence and mortality still represents a critical issue for Public Health authorities with almost 570 thousand new cases and 311 thousand deaths, annually. This is particularly disturbing if considers that the majority of these cases are found in developing and poor countries of Africa and South America [1]. The limitations of screening effectiveness in poor countries are usually related to the scarcity of qualified human resources, lack of governmental planning for organized screening programs, lower quality control in cytology performance and absence of HPV testing, in addition to other reasons generally related to the lower economic incoming to support prevention cancer programs. This set of variables represents a huge barrier in the eradication of a potentially preventable malignancy that affects mostly women living in rural and remote areas and accentuates ethnical and racial inequities regarding the opportunities to reduce cancer mortality [2,3].

HPV vaccination is a well-tailored option for primary prevention tool of HPV-related cancer. Currently, a number of evidences strongly suggest that HPV vaccination is an important implement to reduce the prevalence of malignancies induced by persistent HPV oncogenic types infection in women underprivileged of medical assistance. Recently, an elegant report conducted by Hall and colleagues indicates the possibility of cervical cancer eradication within the next 20 years in Australia by the high-coverage vaccination and screening. The aim is to decrease the rates to four new cases per 100,000 women per year, to consider cervical cancer as an extinguished problem of public health in Australia [4]. Arbyn and co-workers at Cochrane publication demonstrated the efficacy of HPV vaccination, which the data available about HPV vaccine have shown a high-certainty evidence of the protective effect of HPV vaccines against cervical intraepithelial neoplasia in female adolescents and young women aged 15 to 26 [5].

However, despite to the scientific encouragement towards vaccine implementation, there are many restrictive elements for HPV vaccines application with evident reluctance in many settings of the general population, which represent a serious obstacle for HPV-induced lesions reduction, which must be overcomes to increase the vaccine coverage that comprise psychological, religious and/or cultural reactions [6].

The report of Muñoz and colleagues defined with robust data from different countries the most prevalent HPV types worldwide [7]. This is relevant because HPV vaccines were tailored based on the most important HPV types related to cervical cancer development, and the results achieved clearly demonstrated the significant contribution of these vaccines to reduce HPV-related lesions in women cervix [[8], [9], [10]]. Conversely, some HPV types distribution are unequal worldwide, and these variations can induce alterations on the efficacy of HPV vaccine [11]. Moreover, some specific HPV types present to biological behavioral differences that can correspond to better or worse outcome for the women affected with cervical cancer [12].

Considering all these facts, we sought to investigate specific HPV types prevalence in rural and remote areas of Brazilian territory in order to draw a map of HPV types distribution in a country with continental dimension.

## 2 Methods

This was a retrospective evaluation of individual HPV types prevalence performed within a casuistic planned for a prospective study carried out from March to December 2010 of women attended at the Ambulatory of Prevention Department and the mobile units of Barretos Cancer Hospital (BCH), which assisted the Brazilian rural and remote areas, and these samples were previously underwent to Surepath Liquid-based Papanicolaou, and the HPV detection was performed using the careHPV™ molecular test [3].

The project was approved by the Research Ethics Committee of the Barretos Cancer Hospital - Pio XII Foundation, protocol number 404/2010.

### 2.1 The mobile units platform

A total of 3079 volunteer women aged over than 18 were invited to participate in a opportunistic screening available at the mobile units of the Barretos Cancer Hospital, which assisted the states of Goiás, Minas Gerais, Mato Grosso and São Paulo. At this stage, there was no randomization of the studied group and the samples were collected by health professionals (careHPV™ test and Liquid-based Papanicolaou test).

#### 2.1.1 Collections carried out in mobile units

The samples collected to careHPV ™ medium were initially analyzed in the mobile unit in ten out 63 cities visited by the unit: Mineiros (GO), Portelândia (GO), Alto Araguaia (MT), Alto Garças (MT), Alto Taquari (MT), Rondonópolis (MT), Pedra Preta (MT), Guiratinga (MT), São José do Povo (MT), Itiquira (MT); totalizing over than 400 samples analyzed *in loco*; the remaining samples were tested at the Research Molecular Center of BCH.

### 2.2 Laboratory conditions for samples preservation

Cervico-vaginal samples tested for careHPV™ (Digene, Qiagen, Gaithersburg, MD, USA) were maintained under at 15 °C to 30 °C for two weeks, and/or 2 °C to 8 °C for four weeks or −20 °C for two months. Those samples that were tested positive in the careHPV test were maintained 2 °C to 8 °C.

### 2.3 DNA extraction

DNA from the positive samples maintained on the careHPV™ medium were extracted using the QIAmp MinElute Virus Vaccum kit (Qiagen Co) according to the manufacturer's protocol. Extracted DNA were kept in at −20 °C.

### 2.4 HPV genotyping

HPV positive samples using the careHPV™ test were further genotyped using the Luminex methodology (Luminex Corporation, TX, USA). This methodology uses oligonucleotide probes coupled polystyrene beads containing two different fluorophores inside that allows the simultaneous detection of at most 100 different specific HPV types. In this study, probes used allowed the identifications of 21 viral types: i) low risk: 6, 11 and 70, ii) high risk: 16, 18, 26, 31, 33, 35, 39, 45, 51, 52, 53, 56, 58, 59, 66, 68, 73 and 82. PCR reactions were performed using the Qiagen Multiplex PCR kit (Qiagen, Dusseldorf, Germany) and a mixture of specific biotinylated primers capable of amplifying a fragment of the E7 gene, followed by bead-based Luminex technology (Gheit et al., 2007; Nunes et al., 2016; Schmitt et al., 2006). Primers for the amplification of β-globin were also added to evaluate the quality of template DNA. For each probe, MFI (mean fluorescence intensity) values obtained when no PCR product was added to the hybridization mixture was considered the background values. Cutoffs were computed by adding 20 MFI to 1.1X the median background value. Whenever a positive signal for HPV and β-globin was not obtained, the sample was considered inadequate. Those cases in which the positive signal for HPV was not observed, but β-globin was detected, samples were considered negative as well.

### 2.5 Statistical analysis

The prevalences of HPV specific types were computed. Statistical analysis was performed using IBM® SPSS® Statistics (Statistical Package for Social Sciences) version 20.1 for Windows (IBM Corporation, Route 100, Somers, NY 10589) and MedCalc® version 11.1 for Windows (Broekstraat 52, B −9030 Mariakerke, Belgium).

## 3 Results

Women tested positive for HPV (10.0% or n=307) using the careHPV test showed mean age of 41.0 years. The socio-demographic findings achieved previously did not show any statistically significant association between HPV positivity and tobacco use, number of parities, number of sexual partners and contraceptive [3].

### 3.1 HPV types

From 443 samples available for analysis, high-risk HPV was detected in 369 samples, 72 were positive for high and low risk HPV simultaneously and 2 cases tested positive for low risk HPV (both HPV6).

Single HPVs infections were observed in 25.3% (112/443) of the samples, two of them by low-risk HPV (HPV 6). Conversely, 74.7% (331/443) of the samples had multiple infections solely with high-risk types or with high- and low-risk HPVs.

**Fig. 1:**
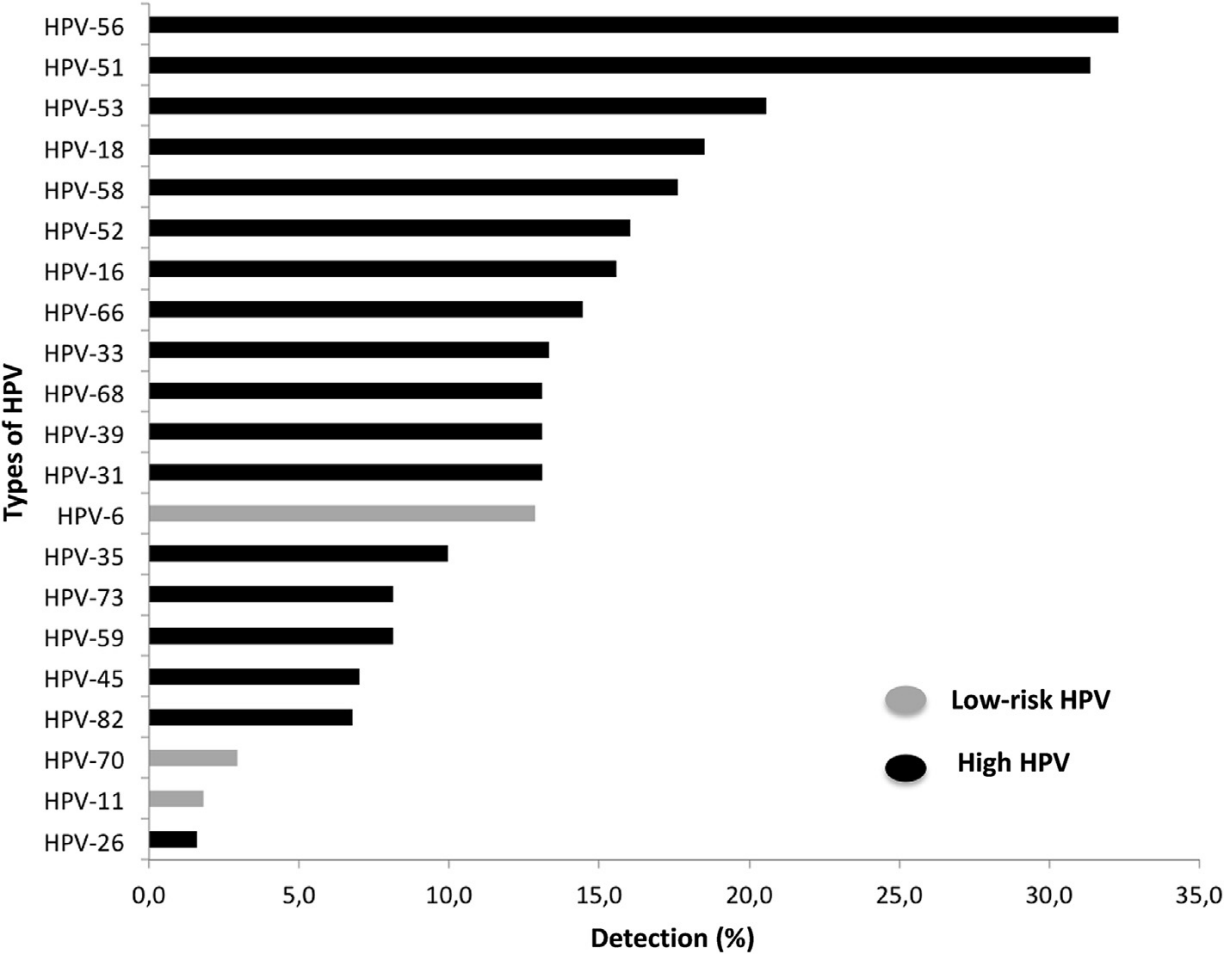
General distribution of HPV genotypes detected in the cervicovaginal samples. The different genotypes are marked on the “Y" axis, while the frequency of the cases (shown in percentage) are on the “X" axis.

HPV-56 and HPV-51 were the most common types, and were detected in 32.3% and 31.4% of the samples, respectively. This represents 3.7% of HPV prevalence, considering the percentage of HPV positive women found in this study (11.5%). Following HPV-53 (20.5%), HPV-18 (18.5%), HPV-58 (17.6%), HPV-52 (16.0%) and HPV-16 (6%) were the most frequent, which represents a prevalence of 2.35%, 2.12%, 2.02%, 1.84% and 1.80%, respectively, according to the percentage of HPV positive women studied. Regarding low-risk HPVs, HPV-6 type was detected in about 12.9% of the samples. The less frequent detected types (<3%) were: HPV-70, HPV-11 and HPV-26. Fig. 1 depicts HPV types distribution.

## 4 Discussion

The general positivity for high-risk HPV reported in this current study was 10.6%: 11.5% observed in the casuistic of cases collected at the Prevention Department of HCB and 10% detected in women examined at mobile units, respectively. Data documented by other Brazilian studies showed positive rates ranging from 9.7% to 10.5% [[13], [14], [15], [16], [17]]. Recently, Torres and colleagues reported HPV-DNA frequency of 19.1% in women of remote areas of the Amazon, a region reputed as the site with the highest indexes of HPV prevalence in the Brazilian territory [18]. The HPV prevalence found in the current study was, however, slightly lower than those found in previous studies, with a prevalence superior to 15% [19] and quite similar to the prevalence of HPV infection of 9.6% found in Malasy population [20].

HPV genotyping performed within the positive samples showed in descending order, higher frequencies of the HPV-53, HPV-18, HPV-58, HPV-52, and HPV-5 and HPV- HPV-16, with frequencies of detection of 32.3%, 31.4%, 20.5%, 18.5%, 17.6%, 16.0% and 15.6%, respectively. Moreover, the overall HPV typing demonstrated that HPV types frequencies is particularly different if compared to the frequencies reported in literature, once HPV56, HPV51 and HPV53 were significantly more prevalent than HPV 16 and HPV18, presumed as the more prevalent HPV types worldwide [7]. Interesting, however, is that fact that previous studies, including a Canadian study, have reported a HPV DNA detection rate of 1 to 7% of HPV-56 in CIN2 and CIN3 patients [[21], [22]]. In a retrospective study in Brazilian Bahia State, about 14% of women with high-grade lesions and 18% with low-grade lesions were identified with HPV-16 and HPV-56 infection [23]. These data differ substantially from another study carried out in the Northern region of Brazil that place HPV-16 and HPV-18 as the most prevalent types in women infected by HPV [24]. We speculate that these variations in prevalence of HPV types are certainly particular to each different region within the Brazilian territory; once this is a country of continental dimensions and formed by a complex network of different cultures and races, which may present more variations regarding HPV type regionally distribution. However, it is important to emphasize that the distribution of different HPV types is not homogeneous worldwide. In this field, HPV16 and HPV18 still show an important and significant protagonism even in Brazil [25]. Some studies have demonstrated that the five most common HPV types in the order of decreasing prevalence are HPV 16, 18, 31, 45 and 52 [26].

In addition, it is necessary to note that differences in the methodologies used to detect and type HPVs can certainly influence data of the different studies regarding HPV types prevalence.

Finally, the importance of HPV typing seems to be closed related to the contribution that these observations can provide to the strategies of vaccination in different regions of the world. This issue is not negligible if one takes in account that HPV vaccination should be planned for certain regions of the world, considering that the variation of HPV typing could infer a bias of HPV vaccine efficacy if the most prevalent types are not included among types selected for bivalent, quadrivalent or nonavalent vaccine currently available in the medical armamentarium. Cross-protection is presently and potentially expectable for all of the 3 licensed HPV vaccines provide protection against HPV types not included in the vaccines. It is well-stablished that bivalent and quadrivalent HPV vaccines provide some level of cross-protection against high-risk HPV types: 31, 33 and 45 HPV types. However, the degree of cross-protection against HPV types of nonavalent vaccine is not yet documented [27].

In conclusion, our study demonstrated that HPV types have a significative variation prevalence in Brazilian territory; data can be improved with robust increasing casuistry.

## Disclosure

The careHPV ™ test kits, as well as equipment, preservation media, consumables and brushes for molecular testing, were provided by Qiagen® Brazil.

## Funding statement

The study was supported financially (grant-aid) of the National Institute of Research and Technology in HPV (INCT - HPV) of São Paulo, through the CNPq process nº 573799/2008-3, FAPESP process nº 2008/57889-1, through and CNPq process nº 203348/2014-1.
